# Deciphering the causal influence of BMI and related metabolic, inflammatory, and cardiovascular factors on brain structure: a Mendelian Randomization Study

**DOI:** 10.1038/s41380-026-03501-x

**Published:** 2026-03-09

**Authors:** Jodie N. Painter, Alexander Refisch, Moritz Rau, Martin Walter, Scott Mackey, Jennifer Laurent, Paul M. Thompson, Katrina L. Grasby, Tomas Hajek, Sarah E. Medland, Nils Opel

**Affiliations:** 1 Brain and Mental Health Program, QIMR Berghofer, Herston, QLD Australia; 2 School of Biomedical Sciences, University of Queensland, St Lucia, QLD Australia; 3 Department of Psychiatry and Psychotherapy, Jena University Hospital, Jena, Germany; 4 Center for Intervention and Research on adaptive and maladaptive brain Circuits underlying mental health (C-I-R-C), Jena-Magdeburg-Halle, Jena, Germany; 5 German Center for Mental Health (DZPG), Jena, Germany; 6 Department of Psychiatry, University of Vermont, Burlington, VT USA; 7 Department of Nursing, University of Vermont, Burlington, VT USA; 8 University of Southern California Keck School of Medicine, Imaging Genetics Center, Mark and Mary Stevens Neuroimaging and Informatics Institute, Los Angeles, California USA; 9 School of Biomedical Sciences, Queensland University of Technology, Herston, QLD Australia; 10 Department of Psychiatry, Dalhousie University, Halifax, NS Canada; 11 Department of Psychiatry & Neuroscience, Campus Benjamin Franklin, Charité Universitätsmedizin, Berlin, Germany; 12 German Center for Mental Health (DZPG), Partner Site Berlin-Potsdam, Berlin, Germany

**Keywords:** Neuroscience, Diagnostic markers

## Abstract

Obesity is a highly prevalent metabolic risk factor that commonly coincides with additional metabolic, cardiovascular, and inflammatory abnormalities. Obesity has frequently been shown to affect brain physiology at multiple levels, and to increase the risk for the development of neuropsychiatric disorders such as major depression and dementia. Previous large-scale neuroimaging research has consistently shown overlapping brain structural alterations in obesity and neuropsychiatric disorders, with the most pronounced alterations being lower cortical thickness in the frontal and temporal cortex. Yet, the direction of association, and the potential causal effect of obesity on brain structural decline, remains unclear. Moreover, it is imperative to determine which of the multifaceted biological systems impacted by obesity, encompassing metabolic, cardiovascular, and inflammatory aspects, may be implicated in the link between obesity and brain structural decline. In this study, we employed univariate and multivariate Mendelian randomization (MR) as an instrumental variable (IV) approach to clarify the causal direction of the relationship between body mass index (BMI) and brain structure and to disentangle the metabolic, cardiovascular, and inflammatory factors that might underlie this relationship. We found evidence for a potential causal influence of elevated BMI on lower cortical thickness, with the most prominent effects in the precentral and fusiform gyrus. We furthermore found concurrent nominal associations of visceral adipose tissue (VAT) and the inflammatory serum marker CRP with lower cortical thickness which largely overlapped with regions associated with higher BMI. In contrast, very few associations with cortical thickness emerged for blood pressure or metabolic serum markers. Our findings thus corroborate the notion of a causal effect of BMI on lower cortical thickness. Future research should aim to delineate if and how the BMI related effect on brain structural decline conveys an increased risk for the development of neuropsychiatric disorders.

## Introduction

Obesity is a hereditary condition [1] that commonly coincides with a spectrum of metabolic, cardiovascular, and inflammatory disturbances. Specifically, elevated blood pressure, impaired glucose metabolism, excess visceral fat accumulation, and alterations in serum triglycerides and cholesterol levels are prevalent comorbid health issues linked to obesity, collectively characterizing the metabolic syndrome [2, 3]. Furthermore, adipose tissue secretion of pro-inflammatory cytokines is known to induce low-grade inflammation in the context of obesity. In addition to its impact on body-wide metabolic, cardiovascular, and inflammatory systems, accumulating evidence underscores obesity as a significant determinant of brain health, with frequent associations observed between obesity and substantial and widespread brain structural abnormalities [4–8]. Recent work by the Enhancing Neuroimaging Genetics through Meta-Analysis (ENIGMA) consortium and others has consistently demonstrated that higher BMI (weight [kg]/height^2^ [m]), a proxy for increases in fat mass, is associated with brain structural abnormalities in healthy controls as well as in psychiatric patients, primarily with lower fronto-temporal cortical thickness [5, 6, 9, 10]. As the majority of previous neuroimaging studies investigating the association between obesity and the brain were based on cross-sectional designs, the direction of associations between higher BMI and brain structural alterations is unknown. Most importantly, it is unclear whether brain structural alterations precede and predispose to higher BMI or if higher BMI induces changes in brain structure.

As previous reports have found that genetic variants associated with BMI are highly expressed in the central nervous system (CNS) [11], it appears possible that genetically determined alterations in brain structure might precede weight gain and hence mediate the effect of genetic risk on the development of obesity. This notion is supported by findings from prospective functional MRI studies reporting that changes in reward system signalling might predict future weight gain [12, 13]. In an attempt to address this open research question, previous imaging genetic research has revealed associations between polygenic risk for BMI and alterations in brain structure, particularly prefrontal grey matter volume reductions and reduced occipital surface area [6, 14].

However, the previously observed associations between polygenic risk for obesity and brain structure are of small effect size and limited to specific brain regions such as the medial prefrontal cortex [6]. In contrast, the replicated phenotypic associations between BMI and brain structure, which have been extensively validated, are broadly distributed and have effect sizes that exceed those observed for common neuropsychiatric disorders [6]. A large proportion of the BMI-related variation in brain structure thus cannot be directly traced back to genetic variants. Furthermore, previous longitudinal studies have reported increased BMI to be associated with decreased grey matter volume and cortical thickness decline over time, thus underpinning the notion of potential BMI-related atrophic or neurodegenerative processes [15, 16]. This is in line with recent reports on accelerated brain ageing in individuals with overweight or obesity [17, 18].

With regard to the potential biological mechanisms underlying the association between BMI and brain structural alterations, a growing body of data suggests chronic low-grade inflammation to represent a fundamental common biological mechanism linking obesity and neuropsychiatric disorders [19]. Since adipose tissue expansion upon a high-fat diet results in a disruption of an anti-inflammatory milieu with increased differentiation and recruitment of pro-inflammatory immune cells [20, 21], chronic low-grade inflammation may be attributed to increased BMI. By promoting oxidative stress and dysregulation in energy-regulating neuroendocrine metabolic pathways, chronic low-grade inflammation contributes significantly to impaired energy supply, to which the CNS is highly vulnerable [22]. Beyond that, emerging evidence indicates cross-talk between systemic inflammatory processes and inflammation in the CNS, further exacerbating neuronal injury as a potential source for the manifestation of neuropsychiatric symptoms [23].

One possibility to investigate the direction of associations between BMI and brain structure are instrumental variable (IV) based methods such as Mendelian Randomization (MR). Utilizing summary statistics from large-scale genome-wide association studies (GWAS), MR offers the possibility to investigate whether observational associations between exposures and outcomes are consistent with causal effects, as genetic variants are fixed at conception, and unlikely to be impacted by confounding or reverse causation. MR has previously been successfully applied to clarify causal relationships between BMI and observationally-associated traits [24–26]. Multivariate MR analyses provide a valuable tool for unravelling complex relationships among a multitude of associated traits, particularly essential in the context of obesity and its multifaceted interactions with metabolic, cardiovascular, and inflammatory factors.

Using summary statistics from large-scale GWAS for BMI, related metabolic, cardiovascular and inflammatory traits and cortical structure, we present an MR study as a hypothesis-driven instrumental variable approach to clarify the causality and direction of the relationship between BMI and cortical thickness across the entire brain and to disentangle the metabolic, cardiovascular, and inflammatory factors that might underlie this relationship. Based on the frequently replicated observational association between higher BMI and lower cortical thickness, particularly in the frontal and temporal cortices, our principal hypothesis posits a putative causal effect of higher BMI on lower cortical thickness primarily in the frontal and temporal cortex. Second, we hypothesize that low-grade inflammation in addition to further metabolic and systemic parameters could be associated with BMI related cortical thickness alterations.

## Methods

### GWAS summary statistics for mendelian randomization analyses

Summary statistics for the construction of an IV for BMI as the primary exposure were taken from the combined Genetic Investigation of Anthropometric Traits (GIANT) Consortium [11] and UK Biobank GWAS meta-analysis that included up to 681,275 individuals of European descent [27] (Supplementary Table 1). Summary statistics for the construction of IVs for the additional exposures investigated here were taken from GWAS for visceral adipose tissue (VAT) as a measure of fat stored around internal organs [28], C-reactive protein (CRP) as a signature marker for chronic inflammation [29], blood pressure measures (systolic blood pressure (SBP), diastolic blood pressure (DBP) and pulse pressure (PP) [30]), serum fasting glucose (FG) [31], high-density lipoprotein (HDL) [32] and triglycerides (TG) [33] (Supplementary Table 2).

Primary neuroimaging outcomes were cortical thickness measures across Desikan–Killiany parcels. We did not include subcortical volumes as confirmatory endpoints because prior large-scale multi-site studies report more consistent effects for cortical thickness (notably lower frontal and temporal cortical thickness), whereas subcortical associations are heterogeneous in direction [5, 6, 9, 10]. Moreover, thickness and volume reflect distinct biological constructs and require different normalization (e.g., ICV) and error-control strategies; restricting analyses to thickness ensured a theoretically coherent and statistically well-controlled test of our a priori hypotheses. The corresponding GWAS summary statistics for cortical thickness measured globally and at 34 regions of interest (ROIs) as the outcomes were taken from the ENIGMA Consortium GWAS meta-analyses of Grasby et al. [34]. Using genetic and brain MRI data for up to 23,183 individuals of European ancestry, these meta-analyses were conducted for average thickness of the entire cortex (global average thickness) and for hemisphere-averaged cortical thickness for the 34 ROIs as defined by the Desikan-Killiany cortical atlas [35]. Cortical thickness effect sizes for variants included in all exposure IVs were taken from the GWAS meta-analyses including only the ENIGMA cohorts (*i.e*., excluding the UK Biobank), and run with no genomic-control or correction for global average thickness for the regional analyses [34] (Supplementary Tables 1 and 2). This is in line with observational analyses of directly measured cortical thickness that do not apply global corrections, and allowed us to minimize potential bias due to overlapping samples (primarily from the UK Biobank) in exposure and outcome GWAS [36]. The full ENIGMA genetic summary data are available upon request (http://enigma.usc.edu/research/download-enigma-gwas-results/). Ethics approval for this study was granted by the QIMR Berghofer Ethics Committee (approval: P2204). Participants in all cohorts that contributed to the cortical thickness meta-analysis results used in this study gave written informed consent, with approval granted by local research ethics committees or Institutional Review Boards [34].

### Instrumental variable construction

For each exposure, an IV was constructed using only variants reaching the genome-wide significant threshold (P < 5.0 × 10^−08^) in the respective GWAS meta-analysis for that trait (see above). Variants with allele mismatches between exposure and outcome summary data, and with palindromic alleles with minor allele frequencies >0.45, were excluded. Linkage disequilibrium between genetic variants was calculated to ensure only independent variants (r^2^ < 0.001, minimum distance 10,000 Kb) were included in each IV. Instrument strength was determined by calculating F-statistics [37] and I^2^_GX_ [38] (with strong instruments shown by values > 10, and close to 1, respectively) for each IV. To enable comparison of effect sizes across traits and brain regions, GWAS effect sizes were standardized using the formula β = B/standard deviation of the estimated trait variance explained by the variants included in each IV for all exposures and outcomes [39] (Supplementary Tables 3 & 4). Results from analyses utilising standardised β are reported in the main text, while results from analyses utilising unstandardised B are included in Supplementary Tables.

### Statistical analyses

Univariate two-sample MR analyses were conducted using the TwoSampleMR program [39]. For each exposure (e.g. BMI etc)-outcome (cortical thickness) analysis, association effect sizes were calculated using the inverse variance weighted (IVW) MR method as the primary analysis. As IVW-MR assumes all exposure-associated variants are valid IVs, we also performed sensitivity analyses using methods that allow for the presence of some invalid variants, namely the weighted median [40], weighted mode [41] and MR Egger [42] methods. Heterogeneity between variant effect sizes for exposure and cortical thickness traits was investigated using Cochrane’s Q, the direction of causality was inferred using MR Steiger [39], and the presence of directional horizontal pleiotropy was assessed with the MR Egger intercept [43]. To ensure the results were not driven by undetected horizontal pleiotropy, analyses were re-run for all cortical regions associated with BMI, and for which pleiotropy or heterogeneity was detected, using the Mendelian Randomization Pleiotropy RESidual Sum and Outlier (MR-PRESSO) method [44], which removes variants with outlying effect sizes that may indicate associations with confounders.

Multivariable MR (MVMR) was conducted to test the direct effect of associated exposures using the MVMR package [45]. For all pairwise analyses conducted, SNPs associated with one exposure that were not present in the available dataset for the other exposure were replaced by proxy SNPs where possible (r^2^ > 0.08, non-palindromic SNPs only). As this resulted in IVs comprising fewer SNPs than were included in the univariate analyses for most traits, F-statistics and I^2^_GX_ were recalculated to test the strength of these smaller instruments.

The analytical protocol was divided into four steps, which were performed as follows:

1. First, to test our hypothesis of a putative causal effect of higher BMI on lower global cortical thickness as suggested by observational studies, we performed the MR analyses for average thickness of the entire cortex (global thickness).
2. Second, complementary to the global analyses, we conducted MR analyses to assess the putative direction of effect between BMI and 34 specific cortical ROIs defined according to the Desikan-Killiany cortical atlas [34, 35].
3. Third, we performed univariate MR analyses to test whether additional obesity-related metabolic, cardiovascular, and inflammatory traits would show similar associations with cortical thickness globally and regionally.
4. Lastly, for brain regions where cortical thickness was significantly associated with BMI we performed multivariable MR to investigate the influence of cardiovascular, metabolic and/or inflammatory traits on these associations.

### Multiple testing correction

To determine the multiple correction testing threshold across all analyses we used matrix spectral decomposition [46] to account for correlation between the cortical regions and estimate the number of independent variables tested. As this method requires individual level phenotypic data, we used the cortical region phenotypic data from the UK Biobank cohort that contributed to the main (all-cohorts) cortical GWAS meta-analyses presented in Grasby et al [34]. To be consistent with the GWAS meta-analyses, the cortical regions were residualised for biological sex, age, and ancestry. Across the 35 cortical regions included here (global cortex and 34 ROIs) the effective number of traits was estimated to be 29, hence we applied a significance threshold of *P* ≤ 1.7 × 10^−3^. Accordingly, we present results surpassing this threshold as the primary findings for all analyses. For transparency, we also report results at P < 0.05 to characterize effect-size patterns and enable cross-trait comparisons, consistent with our prior work [6, 47].

## Results

### BMI and average thickness of the entire cortex

**Fig. 1:**
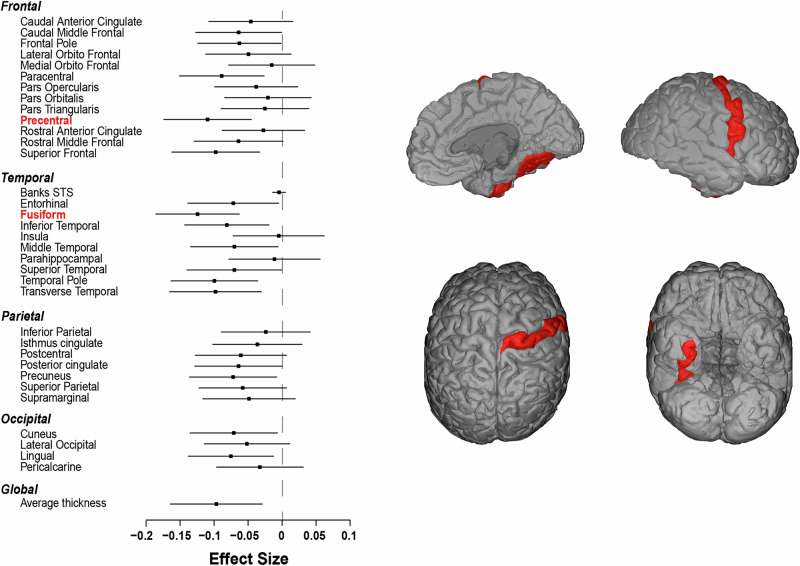
Mendelian randomization testing relationships between body mass index and cortical thickness across 34 cortical regions in addition to global average thickness. As an effect size, the beta values of the calculated Inverse weighted variance were used with error bars showing 95% confidence intervals. Red colour indicates regions which survived multiple comparisons correction. STS Superior Temporal Sulcus. Created with BrainPainter.

Higher BMI was nominally associated with lower average global cortical thickness (β = ^−^0.097, 95% CI = ^−^0.164-^−^0.029, P = 5.07 × 10^−3^); Fig. 1; Supplementary Tables 5 & 6). This association was similarly observed in the sensitivity analysis using MR_PRESSO in which outlier variants were removed (outlier-corrected β = ^−^0.081, 95% CI = ^−^0.146-^−^0.016, P = 1.47 × 10^−2^; Supplementary Table 7).

### BMI and cortical regions of interest throughout the brain

Analyses testing relationships between BMI and hemisphere-averaged cortical thickness at the 34 ROIs yielded significant associations between higher BMI and lower cortical thickness in the precentral gyrus (β = ^−^0.110, 95% CI = ^−^0.1741-^−^0.0451, P = 8.66 × 10^−4^) and the fusiform gyrus (β = ^−^0.097, 95% CI = ^−^0.1858-^−^0.0627, P = 7.55 × 10^−5^) (Fig. 1; Supplementary Tables 5 & 6).

Descriptive analyses of effect sizes for the association between BMI and all cortical ROIs suggested that higher BMI was nominally associated (P < 0.05) with lower cortical thickness in multiple ROIs across the cortex, predominantly in the temporal (7/10 RIOs), frontal (5/13 ROIs) and occipital (2/4 RIOs) cortices, with fewer associations in the parietal (1/6 ROIs) cortex (Fig. 1, Supplementary Tables 5 & 6). The pattern of these associations was overall consistent with sensitivity analyses accounting for potential confounding due to pleiotropy and effect size heterogeneity, with similar effect estimates in the MR-PRESSO analyses accounting for outliers (see Supplementary Table 7).

### Relationship between metabolic, cardiovascular and inflammatory traits with BMI-associated cortical thickness

**Fig. 2:**
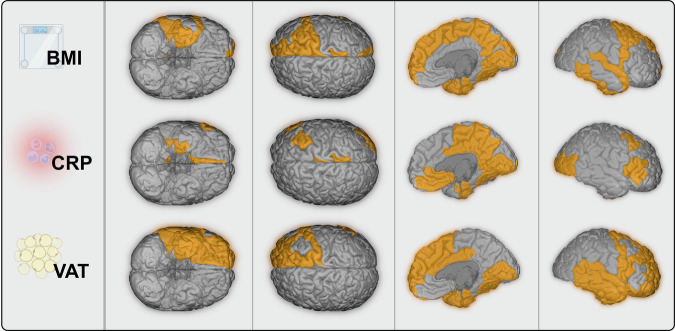
Mendelian randomization testing relationships between additional traits and cortical thickness across cortical regions showing nominally significant negative associations. Orange colour in cortical maps display nominally significant effects in cortical areas associated with body mass index (BMI) and either C-reactive protein (CRP) or visceral adipose tissue (VAT). Beta values of the inverse-variance weighted MR models were used as effect sizes. Created with BrainPainter.

Our follow-up MR analyses of additional metabolic, cardiovascular and inflammatory traits revealed significant associations between higher VAT (β = ^−^0.139, 95% CI ^−^0.2095-^−^0.0681, P = 1.18 × 10^−4^) and higher CRP (β = ^−^0.094, 95% CI ^−^0.1451-^−^0.0426, P = 3.35 × 10^−4^) and lower cortical thickness in the fusiform region (Fig. 2, Supplementary Tables 8 & 9). Higher VAT was also significantly associated with lower cortical thickness in the inferior temporal region (β = ^−^0.1293, 95% CI ^−^0.2062- ^−^0.0525, P = 9.76 × 10^−4^), while higher CRP was significantly associated with lower cortical thickness in the lateral occipital region (β = ^−^0.1024, 95% CI ^−^0.1641-^−^0.0407, P = 1.14 × 10^−3^) (Supplementary Tables 8 & 9).

Nominal associations were also seen across the cortex primarily for higher VAT and higher CRP and lower cortical thickness in frontal and temporal cortical regions. Higher VAT was nominally associated with lower cortical thickness globally and in 17/34 cortical ROIs, mostly overlapping regions nominally associated with BMI (11/16 ROIs) and with broadly similar effect sizes (Fig. 2, Supplementary Tables 5 & 8). Higher CRP was nominally associated with lower cortical thickness globally, and in 14/34 cortical ROIs, almost half of which (7/16 ROIs) were also nominally associated with BMI, VAT or both adiposity measures (Fig. 2, Supplementary Tables 5 & 8). Nominal associations were also seen for other tested exposure traits at a small number of ROIs across the brain: higher DBP (1 ROI) and higher FG (4 ROIs) were nominally associated with lower cortical thickness, while higher PP (1 ROI) and higher HDL (6 ROIs) were nominally associated with higher cortical thickness (Supplementary Tables 8 & 9).

### Multivariable MR

Multivariate MR (MVMR) analyses to test for shared effects between BMI and all additional tested traits were conducted for the precentral and fusiform regions, where higher BMI was significantly associated with lower cortical thickness in the univariate MR analyses. For both regions the effects of BMI and VAT were no longer or only nominally significant when both traits were included in the multivariable analyses, suggesting largely shared effects between these adiposity measures (Table 1). BMI effects were slightly attenuated in analyses including CRP and HDL for both regions, and including triglycerides for the precentral gyrus, suggesting the presence of some shared but also unique direct effects of BMI on cortical thickness that are independent of CRP, HDL or triglyceride levels.

**Table 1:** Multivariable MR analyses testing the influence of traits observationally associated with BMI on the effect of BMI on cortical thickness.

	Model							
	IVW - single trait*				IVW – multivariable MR**			
Region and Trait	β	se	t-value	P	β	se	t-value	P
**Precentral**								
BMI	−0.098	0.028	−3.557	4.03E-04	0.060	0.103	0.585	5.59E-01
VAT	−0.107	0.027	−3.984	7.55E-05	−0.163	0.102	−1.594	1.11E-01
BMI	−0.109	0.033	−3.332	9.11E-04	−0.097	0.039	−2.490	1.30E-02
CRP	−0.020	0.009	−2.286	2.26E-02	−0.006	0.010	−0.615	5.39E-01
BMI	−0.130	0.032	−4.014	6.54E-05	−0.130	0.032	−3.996	7.03E-05
SBP	0.014	0.041	0.345	7.30E-01	0.003	0.041	0.064	9.49E-01
BMI	−0.098	0.032	−3.053	2.34E-03	−0.097	0.032	−2.985	2.92E-03
DBP	0.028	0.039	0.717	4.74E-01	0.014	0.039	0.343	7.32E-01
BMI	−0.098	0.032	−3.053	2.34E-03	−0.097	0.032	−2.985	2.92E-03
PP	0.028	0.039	0.717	4.74E-01	0.014	0.039	0.343	7.32E-01
BMI	−0.108	0.031	−3.441	6.13E-04	−0.085	0.033	−2.596	9.64E-03
HDL	0.075	0.024	3.130	1.82E-03	0.054	0.025	2.171	3.03E-02
BMI	−0.086	0.032	−2.664	7.92E-03	−0.076	0.033	−2.327	2.02E-02
Tri	−0.052	0.027	−1.961	5.03E-02	−0.040	0.027	−1.476	1.40E-01
BMI	−0.080	0.025	−3.156	1.68E-03	−0.084	0.025	−3.302	1.02E-03
FG	−0.067	0.072	−0.930	3.53E-01	−0.097	0.072	−1.347	1.79E-01
**Fusiform**								
BMI	−0.123	0.027	−4.564	6.01E-06	0.071	0.101	0.708	4.79E-01
VAT	−0.134	0.026	−5.067	5.26E-07	−0.200	0.100	−2.003	4.56E-02
BMI	−0.124	0.031	−3.944	8.83E-05	−0.139	0.037	−3.743	1.97E-04
CRP	−0.012	0.008	−1.444	1.49E-01	0.008	0.010	0.768	4.43E-01
BMI	−0.139	0.030	−4.596	4.99E-06	−0.141	0.030	−4.643	4.01E-06
SBP	−0.019	0.038	−0.495	6.21E-01	−0.031	0.038	−0.831	4.06E-01
BMI	−0.118	0.031	−3.855	1.25E-04	−0.119	0.031	−3.867	1.19E-04
DBP	0.005	0.037	0.123	9.02E-01	−0.013	0.037	−0.345	7.30E-01
BMI	−0.118	0.031	−3.855	1.25E-04	−0.119	0.031	−3.867	1.19E-04
PP	0.005	0.037	0.123	9.02E-01	−0.013	0.037	−0.345	7.30E-01
BMI	−0.133	0.029	−4.506	7.72E-06	−0.127	0.031	−4.112	4.38E-05
HDL	0.043	0.023	1.890	5.91E-02	0.012	0.024	0.527	5.98E-01
BMI	−0.108	0.030	−3.592	3.52E-04	−0.109	0.031	−3.537	4.32E-04
Tri	−0.015	0.025	−0.613	5.40E-01	0.002	0.025	0.093	9.26E-01
BMI	−0.083	0.024	−3.429	6.48E-04	−0.086	0.025	−3.488	5.23E-04
FG	−0.017	0.069	−0.251	8.02E-01	−0.048	0.069	−0.694	4.88E-01

Association effects were estimated using IVs that included only SNPs (or proxy SNPs) present in both of the listed exposure traits per analysis.

*The IVW - single trait columns show effect sizes for univariate analyses using the respective IVs per trait.

**The IVW – multivariable MR columns show the direct effects per traits conditioning on the other trait in each multivariate MR analysis.

## Discussion

In the present study, using MR, we provide evidence suggesting a putative causal effect of BMI on cortical thickness across the human brain. The most prominent effects were observed in the precentral and fusiform gyri, with nominal effects observed across the frontal and temporal cortices. We furthermore found evidence for concurrent associations of higher VAT and CRP with lower cortical thickness most prominently in the fusiform gyrus, with nominal evidence for further associations across the brain and largely overlapping regions where lower cortical thickness was also associated with higher BMI.

Our finding of a significant and nominal associations between higher BMI and lower cortical thickness particularly in the temporal cortex is in line with findings from the latest large-scale multicenter analyses [10, 48] that congruently report a relationship between higher BMI and lower temporal cortical thickness. In fact, the most up-to-date and largest mega-analysis from the ENIGMA consortium based on 6420 participants indicated the strongest regional effect sizes for the association between higher BMI and lower cortical thickness in the fusiform gyrus [6], supported by the findings of our MR analyses. More recent large-scale studies by the ENIGMA consortium and others further corroborate the association between higher BMI and lower temporal thickness [9, 49].

Two separate MR studies have also recently presented evidence for an impact of obesity on cortical thickness [50, 51]. Limitations in both studies, however, resulted in smaller numbers of associations seen across the brain. Further, in the case of Chen et al. [51], there is evidence for both lower and higher cortical thickness with higher BMI. Both studies focused only on adiposity and included regional thickness data that had been corrected for global thickness; in the current study we used summary statistics that had not undergone such correction, in line with analyses in observational studies, to interrogate region-specific impacts [6]. Incorporating these considerations, it is apparent that such differences in methodology would have led to very different interpretations. Additionally, Chen et al. [51] used GWAS summary statistics for both the adiposity and cortical measures that were generated including individuals from the UK Biobank, which is likely to have biased their results due to the large number of overlapping samples in the exposure and outcome datasets [51].

The main findings of our hypothesis-driven study hold several relevant implications for future research. First, our genetic results support the hypothesis of a potentially causal effect of BMI in brain structural decline through an effect on cortical thickness. Importantly, this is consistent with previous findings from longitudinal human neuroimaging studies reporting BMI-related brain structural atrophy over time [15] as well as with recent reports on accelerated brain ageing in obesity [5, 52].

Our findings also raise the question of the biological mechanisms underlying the effect of BMI on cortical structural decline. In this regard, our finding of parallel associations of VAT and CRP with lower cortical thickness that largely corresponded with BMI-associated changes across the frontal and temporal cortices, appears noteworthy. Although the majority of these associations did not surpass our stringent multiple-testing threshold, our findings align with prior research linking obesity, inflammation, and altered brain physiology globally and in cortical regions in particular [53–55]. Notably, past studies have emphasized the role of VAT in generating pro-inflammatory cytokines, potentially exerting adverse effects on the CNS. More specifically, pro-inflammatory molecules released by lymphocytes and M1 macrophages residing in the VAT were shown to induce apoptosis through microglia stimulation inside of the CNS, a possible mechanism for cortical thinning [56–58]. While our current MVMR results indicated little influence of CRP on the direct effect of BMI on cortical thickness, our combined results underscore the need for future mechanistic investigations in this domain.

In addition to the univariate findings for VAT and CRP, it should be noted that none of the other examined factors, including glucose metabolism, serum triglycerides, lipoproteins and blood pressure, reached statistical significance or exhibited a consistent pattern of associations with cortical thickness across the brain. This finding is striking considering previous biological research suggesting a potential connection between the vascular system and brain integrity including findings of associations between higher BMI and white matter hyperintensities, which are known to be related to cerebrovascular diseases [59]. While clarification of the mechanistic underpinnings between the putative causal link between BMI and cortical thickness will rely on preclinical research (e.g., animal models), future translational research should take advantage of methodological progress in neuroimaging to clarify the relationship between BMI and brain structural decline. Here, investigation of perivascular spaces and glymphatic clearance through ultra-fast magnetic encephalography represent promising approaches for the investigation of intermediate phenotypes related to neuroinflammation and neurovascular changes in obesity in-vivo [60, 61].

While the overwhelming relevance of obesity and related metabolic dysregulation as a cardiovascular risk factor has long been recognized by research and clinical practice, our results advocate for the relevance of higher BMI as a risk factor for the development of neurodegenerative disorders. Higher BMI has been shown to be an important risk factor for several neurodegenerative and psychiatric disorders and has recently been stated to be the most important modifiable risk factor for dementia in the USA [62, 63]. The putative causal effect of higher BMI on lower cortical thickness observed in the present study appears to be in line with the aforementioned reports. It could thus be speculated that lower cortical thickness might act as a relevant mediator in the association between adverse metabolic conditions and neuropsychiatric disorders. Atrophy of brain regions located in the temporal lobe has long been known to be associated with the subsequent development of cognitive impairment and dementia [64–66]. Future studies should thus aim to further clarify the role of BMI-related thickness decline in the fusiform temporal and precentral frontal gyrus as the most prominent findings in the present study, in the development of neuropsychiatric disorders.

This study has a number of strengths and limitations. Strengths include the application of MR analyses to test hypotheses based on previous observational studies, using genetic information from large-scale meta-analyses of BMI as well as a comprehensive investigation of genetic determinants influencing cortical thickness. These data allowed us to investigate both global as well as regional specific associations between BMI and cortical thickness, with evidence of causal associations between BMI and brain structure complementing previous cross-sectional and longitudinal neuroimaging studies. The regional specific analyses allowed us to confirm a putative causal effect of BMI particularly in the fusiform gyrus, along with suggestive evidence in other ROIs in the temporal cortex, thus providing candidate brain regions for subsequent mechanistic studies on underlying biological mediators. The inclusion of a variety of relevant related traits covering a broad spectrum of metabolic, cardiovascular and inflammatory traits represents another strength as it allowed to disentangle and identify potential candidate systems for future mechanistic research on the impact of BMI on brain health.

Limitations to our study should also be acknowledged. First, while we were able to investigate associations between BMI and cortical thickness, we could not investigate whether the thickness of the cortex globally or in particular regions influences BMI due to the current paucity of cortical thickness-associated genetic variants (10 genome-wide significant loci across all 34 ROIs). The power of our analyses may have been impacted by the current relatively low levels of genetic variance in cortical thickness explained by GWAS variants [35], and by the reduced numbers of overlapping variants included in the IVs utilised in the multivariate analyses, limiting our investigations of the influence of cardiovascular, metabolic and inflammatory traits on BMI-associated cortical thickness. We did not examine subcortical morphology in the primary analyses. Given heterogeneous prior findings for subcortical volumes and their differing methodological requirements relative to thickness, we prioritized a focused cortical thickness-based analysis. Further, current data do not allow us to infer the exact timing of potential causal effects of BMI on lower cortical thickness. It thus remains unclear during which developmental stages and at which speed increased BMI might cause brain structural decline. Future larger GWAS meta-analyses, particularly for cortical thickness, and longitudinal cohort studies are warranted to address these open research questions.

Collectively, the results of the present study corroborate the notion of a putative causal effect of BMI on brain structural volume decline through lower cortical thickness. The pattern of increased BMI associated with lower cortical thickness calls for increased attention towards the relevance of obesity and related metabolic conditions as modifiable risk factors for brain health. These findings highlight the need for future experimental investigations aimed at unravelling the potential cascade of mechanisms and identifying intervention opportunities within the connection between weight gain, adipose tissue and structural brain changes. Future research and preventive efforts should aim to further explore the biological mechanisms through which BMI might influence brain structural decline and clarify the relationship between BMI related brain structural impairment and specific domains of neurocognitive functioning.

## Supplementary information

Supplementary Material

## References

[1] Bouchard C. Genetics of obesity: what we have learned over decades of research. Obesity. 2021;29:802–20. 10.1002/oby.23116 33899337 10.1002/oby.23116

[2] Eckel RH, Grundy SM, Zimmet PZ. The metabolic syndrome. Lancet. 2005;365:1415–28. 10.1016/s0140-6736(05)66378-7 15836891 10.1016/S0140-6736(05)66378-7

[3] Powell-Wiley TM, Poirier P, Burke LE, Després JP, Gordon-Larsen P, Lavie CJ, et al. Obesity and cardiovascular disease: a scientific statement from the American heart association. Circulation. 2021;143:e984–e1010. 10.1161/cir.0000000000000973 33882682 10.1161/CIR.0000000000000973 PMC8493650

[4] Janowitz D, Wittfeld K, Terock J, Freyberger HJ, Hegenscheid K, Völzke H, et al. Association between waist circumference and gray matter volume in 2344 individuals from two adult community-based samples. Neuroimage. 2015;122:149–57. 10.1016/j.neuroimage.2015.07.086 26256530 10.1016/j.neuroimage.2015.07.086

[5] McWhinney S, Kolenic M, Franke K, Fialova M, Knytl P, Matejka M, et al. Obesity as a risk factor for accelerated brain ageing in first-episode psychosis-a longitudinal study. Schizophr. Bull. 2021;47:1772–81. 10.1093/schbul/sbab064 34080013 10.1093/schbul/sbab064 PMC8530396

[6] Opel N, Thalamuthu A, Milaneschi Y, Grotegerd D, Flint C, Leenings R, et al. Brain structural abnormalities in obesity: relation to age, genetic risk, and common psychiatric disorders: Evidence through univariate and multivariate mega-analysis including 6420 participants from the ENIGMA MDD working group. Mol. Psychiatry. 2021;26:4839–52. 10.1038/s41380-020-0774-9 32467648 10.1038/s41380-020-0774-9 PMC8589644

[7] Repple J, Opel N, Meinert S, Redlich R, Hahn T, Winter NR, et al. Elevated body-mass index is associated with reduced white matter integrity in two large independent cohorts. Psychoneuroendocrinology. 2018;91:179–85. 10.1016/j.psyneuen.2018.03.007 29571075 10.1016/j.psyneuen.2018.03.007

[8] Veit R, Kullmann S, Heni M, Machann J, Häring HU, Fritsche A, et al. Reduced cortical thickness associated with visceral fat and BMI. Neuroimage Clin. 2014;6:307–11. 10.1016/j.nicl.2014.09.013 25379443 10.1016/j.nicl.2014.09.013 PMC4215386

[9] McWhinney SR, Abé C, Alda M, Benedetti F, Bøen E, Del Mar Bonnin C, et al. Diagnosis of bipolar disorders and body mass index predict clustering based on similarities in cortical thickness-ENIGMA study in 2436 individuals. Bipolar Disord. 2022;24:509–20. 10.1111/bdi.13172 34894200 10.1111/bdi.13172 PMC9187778

[10] McWhinney SR, Abé C, Alda M, Benedetti F, Bøen E, Del Mar Bonnin C, et al. Mega-analysis of association between obesity and cortical morphology in bipolar disorders: ENIGMA study in 2832 participants. Psychol. Med. 2023;53:1–11. 10.1017/S0033291723000223 36846964 10.1017/S0033291723000223 PMC10600817

[11] Locke AE, Kahali B, Berndt SI, Justice AE, Pers TH, Day FR, et al. Genetic studies of body mass index yield new insights for obesity biology. Nature. 2015;518:197–206. 10.1038/nature14177 25673413 10.1038/nature14177 PMC4382211

[12] Stice E, Burger KS, Yokum S. Reward region responsivity predicts future weight gain and moderating effects of the Taq1A allele. J. Neurosci. 2015;35:10316 10.1523/JNEUROSCI.3607-14.2015 26180206 10.1523/JNEUROSCI.3607-14.2015 PMC4502268

[13] Winter SR, Yokum S, Stice E, Osipowicz K, Lowe MR. Elevated reward response to receipt of palatable food predicts future weight variability in healthy-weight adolescents. Am. J. Clin. Nutr. 2017;105:781–9. 10.3945/ajcn.116.141143 28228422 10.3945/ajcn.116.141143 PMC5366045

[14] Opel N, Redlich R, Kaehler C, Grotegerd D, Dohm K, Heindel W, et al. Prefrontal gray matter volume mediates genetic risks for obesity. Mol. Psychiatry. 2017;22:703–10. 10.1038/mp.2017.51 28348383 10.1038/mp.2017.51

[15] Bobb JF, Schwartz BS, Davatzikos C, Caffo B. Cross-sectional and longitudinal association of body mass index and brain volume. Hum. Brain Mapp. 2014;35:75–88. 10.1002/hbm.22159 23008165 10.1002/hbm.22159 PMC3615109

[16] Driscoll I, Beydoun MA, An Y, Davatzikos C, Ferrucci L, Zonderman AB, et al. Midlife obesity and trajectories of brain volume changes in older adults. Hum. Brain Mapp. 2012;33:2204–10. 10.1002/hbm.21353 22887828 10.1002/hbm.21353 PMC3419372

[17] McWhinney SR, Abé C, Alda M, Benedetti F, Bøen E, Del Mar Bonnin C, et al. Association between body mass index and subcortical brain volumes in bipolar disorders-ENIGMA study in 2735 individuals. Mol. Psychiatry. 2021;26:6806–19. 10.1038/s41380-021-01098-x 33863996 10.1038/s41380-021-01098-x PMC8760047

[18] Kolenic M, Franke K, Hlinka J, Matejka M, Capkova J, Pausova Z, et al. Obesity, dyslipidemia and brain age in first-episode psychosis. J. Psychiatr. Res. 2018;99:151–8. 10.1016/j.jpsychires.2018.02.012 29454222 10.1016/j.jpsychires.2018.02.012

[19] Milaneschi Y, Simmons WK, van Rossum EFC, Penninx BW. Depression and obesity: evidence of shared biological mechanisms. Mol. Psychiatry. 2019;24:18–33. 10.1038/s41380-018-0017-5 29453413 10.1038/s41380-018-0017-5

[20] Chawla A, Nguyen KD, Goh YP. Macrophage-mediated inflammation in metabolic disease. Nat. Rev. Immunol. 2011;11:738–49. 10.1038/nri3071 21984069 10.1038/nri3071 PMC3383854

[21] Zatterale F, Longo M, Naderi J, Raciti GA, Desiderio A, Miele C, et al. Chronic adipose tissue inflammation linking obesity to insulin resistance and type 2 diabetes. Front. Physiol. 2020;10:1607. 32063863 10.3389/fphys.2019.01607 PMC7000657

[22] Devine MJ, Kittler JT. Mitochondria at the neuronal presynapse in health and disease. Nat. Rev. Neurosci. 2018;19:63–80. 10.1038/nrn.2017.170 29348666 10.1038/nrn.2017.170

[23] Clark C, Richiardi J, Maréchal B, Bowman GL, Dayon L, Popp J. Systemic and central nervous system neuroinflammatory signatures of neuropsychiatric symptoms and related cognitive decline in older people. J. Neuroinflammation. 2022;19:127 10.1186/s12974-022-02473-3 35643540 10.1186/s12974-022-02473-3 PMC9148517

[24] Gill D, Zuber V, Dawson J, Pearson-Stuttard J, Carter AR, Sanderson E, et al. Risk factors mediating the effect of body mass index and waist-to-hip ratio on cardiovascular outcomes: Mendelian randomization analysis. Int. J. Obes. 2021;45:1428–38. 10.1038/s41366-021-00807-4 10.1038/s41366-021-00807-4 PMC8236409 34002035

[25] Holmes MV, Lange LA, Palmer T, Lanktree MB, North KE, Almoguera B, et al. Causal effects of body mass index on cardiometabolic traits and events: a Mendelian randomization analysis. Am. J. Hum. Genet. 2014;94:198–208. 10.1016/j.ajhg.2013.12.014 24462370 10.1016/j.ajhg.2013.12.014 PMC3928659

[26] Marini S, Merino J, Montgomery BE, Malik R, Sudlow CL, Dichgans M, et al. Mendelian randomization study of obesity and cerebrovascular disease. Ann. Neurol. 2020;87:516–24. 10.1002/ana.25686 31975536 10.1002/ana.25686 PMC7392199

[27] Yengo L, Sidorenko J, Kemper KE, Zheng Z, Wood AR, Weedon MN, et al. Meta-analysis of genome-wide association studies for height and body mass index in ∼700000 individuals of European ancestry. Hum. Mol. Genet. 2018;27:3641–9. 10.1093/hmg/ddy271 30124842 10.1093/hmg/ddy271 PMC6488973

[28] Karlsson T, Rask-Andersen M, Pan G, Höglund J, Wadelius C, Ek WE, et al. Contribution of genetics to visceral adiposity and its relation to cardiovascular and metabolic disease. Nat. Med. 2019;25:1390–5. 10.1038/s41591-019-0563-7 31501611 10.1038/s41591-019-0563-7

[29] Said S, Pazoki R, Karhunen V, Võsa U, Ligthart S, Bodinier B, et al. Genetic analysis of over half a million people characterises C-reactive protein loci. Nat. Commun. 2022;13:2198 10.1038/s41467-022-29650-5 35459240 10.1038/s41467-022-29650-5 PMC9033829

[30] Evangelou E, Warren HR, Mosen-Ansorena D, Mifsud B, Pazoki R, Gao H, et al. Genetic analysis of over 1 million people identifies 535 new loci associated with blood pressure traits. Nat. Genet. 2018;50:1412–25. 10.1038/s41588-018-0205-x 30224653 10.1038/s41588-018-0205-x PMC6284793

[31] Chen J, Spracklen CN, Marenne G, Varshney A, Corbin LJ, Luan J, et al. The trans-ancestral genomic architecture of glycemic traits. Nat. Genet. 2021;53:840–60. 10.1038/s41588-021-00852-9 34059833 10.1038/s41588-021-00852-9 PMC7610958

[32] Sudlow C, Gallacher J, Allen N, Beral V, Burton P, Danesh J, et al. UK biobank: an open access resource for identifying the causes of a wide range of complex diseases of middle and old age. PLoS Med. 2015;12:e1001779 10.1371/journal.pmed.1001779 25826379 10.1371/journal.pmed.1001779 PMC4380465

[33] Karczewksi JK, Gupta R, Kanai M, Lu W, Tsuo K, Wang Y, et al. Pan-UK Biobank genome-wide association analyses enhance discovery and resolution of ancestry-enriched effects. Nat. Genet. 2025;57:2408–17. 10.1038/s41588-025-02335-7 40968291 10.1038/s41588-025-02335-7 PMC13192283

[34] Grasby KL, Jahanshad N, Painter JN, Colodro-Conde L, Bralten J, Hibar DP, et al. The genetic architecture of the human cerebral cortex. Science. 2020;367:eaay6690 10.1126/science.aay6690 32193296 10.1126/science.aay6690 PMC7295264

[35] Desikan RS, Ségonne F, Fischl B, Quinn BT, Dickerson BC, Blacker D, et al. An automated labeling system for subdividing the human cerebral cortex on MRI scans into gyral based regions of interest. Neuroimage. 2006;31:968–80. 10.1016/j.neuroimage.2006.01.021 16530430 10.1016/j.neuroimage.2006.01.021

[36] Burgess S, Davies NM, Thompson SG. Bias due to participant overlap in two-sample Mendelian randomization. Genet. Epidemiol. 2016;40:597–608. 10.1002/gepi.21998 27625185 10.1002/gepi.21998 PMC5082560

[37] Burgess S, Thompson SG. Avoiding bias from weak instruments in Mendelian randomization studies. Int. J. Epidemiol. 2011;40:755–64. 10.1093/ije/dyr036 21414999 10.1093/ije/dyr036

[38] Bowden J, Del Greco MF, Minelli C, Davey Smith G, Sheehan NA, Thompson JR. Assessing the suitability of summary data for two-sample Mendelian randomization analyses using MR-Egger regression: the role of the I2 statistic. Int. J. Epidemiol. 2016;45:1961–74. 10.1093/ije/dyw220 27616674 10.1093/ije/dyw220 PMC5446088

[39] Hemani G, Zheng J, Elsworth B, Wade KH, Haberland V, Baird D, et al. The MR-Base platform supports systematic causal inference across the human phenome. Elife. 2018;7:e34408 10.7554/eLife.34408 29846171 10.7554/eLife.34408 PMC5976434

[40] Bowden J, Davey Smith G, Haycock PC, Burgess S. Consistent estimation in mendelian randomization with some invalid instruments using a weighted median estimator. Genet. Epidemiol. 2016;40:304–14. 10.1002/gepi.21965 27061298 10.1002/gepi.21965 PMC4849733

[41] Hartwig FP, Davey Smith G, Bowden J. Robust inference in summary data mendelian randomization via the zero modal pleiotropy assumption. Int. J. Epidemiol. 2017;46:1985–98. 10.1093/ije/dyx102 29040600 10.1093/ije/dyx102 PMC5837715

[42] Bowden J, Davey Smith G, Burgess S. Mendelian randomization with invalid instruments: effect estimation and bias detection through Egger regression. Int. J. Epidemiol. 2015;44:512–25. 10.1093/ije/dyv080 26050253 10.1093/ije/dyv080 PMC4469799

[43] Burgess S, Thompson SG. Interpreting findings from Mendelian randomization using the MR-Egger method. Eur. J. Epidemiol. 2017;32:377–89. 10.1007/s10654-017-0255-x 28527048 10.1007/s10654-017-0255-x PMC5506233

[44] Verbanck M, Chen CY, Neale B, Do R. Detection of widespread horizontal pleiotropy in causal relationships inferred from Mendelian randomization between complex traits and diseases. Nat. Genet. 2018;50:693–8. 10.1038/s41588-018-0099-7 29686387 10.1038/s41588-018-0099-7 PMC6083837

[45] Sanderson E, Davey Smith G, Windmeijer F, Bowden J. An examination of multivariable Mendelian randomization in the single-sample and two-sample summary data settings. Int. J. Epidemiol. 2019;48:713–27. 10.1093/ije/dyy262 30535378 10.1093/ije/dyy262 PMC6734942

[46] Li J, Ji L. Adjusting multiple testing in multilocus analyses using the eigenvalues of a correlation matrix. Heredity. 2005;95:221–7. 10.1038/sj.hdy.6800717 16077740 10.1038/sj.hdy.6800717

[47] Opel N, Goltermann J, Hermesdorf M, Berger K, Baune BT, Dannlowski U. Cross-disorder analysis of brain structural abnormalities in six major psychiatric disorders: a secondary analysis of mega- and meta-analytical findings from the ENIGMA consortium. Biol. Psychiatry. 2020;88:678–86. 10.1016/j.biopsych.2020.04.027 32646651 10.1016/j.biopsych.2020.04.027

[48] McWhinney SR, Brosch K, Calhoun VD, Crespo-Facorro B, Crossley NA, Dannlowski U, et al. Obesity and brain structure in schizophrenia - ENIGMA study in 3021 individuals. Mol. Psychiatry. 2022;27:3731–3737. 10.1038/s41380-022-01616-5 35739320 10.1038/s41380-022-01616-5 PMC9902274

[49] Watanabe K, Kakeda S, Nemoto K, Onoda K, Yamaguchi S, Kobayashi S, et al. Effects of obesity, blood pressure, and blood metabolic biomarkers on grey matter brain healthcare quotient: a large cohort study of a magnetic resonance imaging brain screening system in Japan. J. Clin. Med. 2022;11:2973 10.3390/jcm11112973 35683364 10.3390/jcm11112973 PMC9181611

[50] Chen L, Zhao S, Wang Y, Niu X, Zhang B, Li X, et al. Genetic insights into obesity and brain: combine mendelian randomization study and gene expression analysis. Brain Sci. 2023;13:36 10.3390/brainsci13060892 10.3390/brainsci13060892 PMC10295948 37371369

[51] Chen W, Feng J, Guo J, Dong S, Li R, Ngo JCK, et al. Obesity causally influencing brain cortical structure: a Mendelian randomization study. Cereb. Cortex. 2023;33:9409–16. 10.1093/cercor/bhad214 37328935 10.1093/cercor/bhad214

[52] Ronan L, Alexander-Bloch AF, Wagstyl K, Farooqi S, Brayne C, Tyler LK, et al. Obesity associated with increased brain age from midlife. Neurobiol. Aging. 2016;47:63–70. 10.1016/j.neurobiolaging.2016.07.010 27562529 10.1016/j.neurobiolaging.2016.07.010 PMC5082766

[53] Woo A, Botta A, Shi SSW, Paus T, Pausova Z. Obesity-Related neuroinflammation: magnetic resonance and microscopy imaging of the brain. Int. J. Mol. Sci. 2022;23:8790. 35955925 10.3390/ijms23158790 PMC9368789

[54] Chen K-HE, Lainez NM, Nair MG, Coss D. Visceral adipose tissue imparts peripheral macrophage influx into the hypothalamus. J. Neuroinflammation. 2021;18:140 10.1186/s12974-021-02183-2 34154608 10.1186/s12974-021-02183-2 PMC8218389

[55] Opel N, Cearns M, Clark S, Toben C, Grotegerd D, Heindel W, et al. Large-scale evidence for an association between low-grade peripheral inflammation and brain structural alterations in major depression in the BiDirect study. J. Psychiatry Neurosci. 2019;44:423–31. 10.1503/jpn.180208 31304733 10.1503/jpn.180208 PMC6821515

[56] Chen B, Zhong X, Zhang M, Mai N, Wu Z, Chen X, et al. The additive effect of late-life depression and olfactory dysfunction on the risk of dementia was mediated by hypersynchronization of the hippocampus/fusiform gyrus. Transl. Psychiatry. 2021;11:172 10.1038/s41398-021-01291-0 33731679 10.1038/s41398-021-01291-0 PMC7969612

[57] Gómez-Apo E, Mondragón-Maya A, Ferrari-Díaz M, Silva-Pereyra J. Structural brain changes associated with overweight and obesity. J. Obes. 2021;2021:6613385 10.1155/2021/6613385 34327017 10.1155/2021/6613385 PMC8302366

[58] Nguyen JC, Killcross AS, Jenkins TA. Obesity and cognitive decline: role of inflammation and vascular changes. Front. Neurosci. 2014;8:375 10.3389/fnins.2014.00375 25477778 10.3389/fnins.2014.00375 PMC4237034

[59] Lampe L, Zhang R, Beyer F, Huhn S, Kharabian Masouleh S, Preusser S, et al. Visceral obesity relates to deep white matter hyperintensities via inflammation. Ann. Neurol. 2019;85:194–203. 10.1002/ana.25396 30556596 10.1002/ana.25396 PMC6590485

[60] Kiviniemi V, Wang X, Korhonen V, Keinänen T, Tuovinen T, Autio J, et al. Ultra-fast magnetic resonance encephalography of physiological brain activity - Glymphatic pulsation mechanisms? J. Cereb. Blood Flow. Metab. 2016;36:1033–45. 10.1177/0271678x15622047 26690495 10.1177/0271678X15622047 PMC4908626

[61] Wardlaw JM, Benveniste H, Nedergaard M, Zlokovic BV, Mestre H, Lee H, et al. Perivascular spaces in the brain: anatomy, physiology and pathology. Nat. Rev. Neurol. 2020;16:137–53. 10.1038/s41582-020-0312-z 32094487 10.1038/s41582-020-0312-z

[62] Slomski A. Obesity is now the top modifiable dementia risk factor in the US. JAMA. 2022;328:10–10. 10.1001/jama.2022.11058 35788805 10.1001/jama.2022.11058

[63] Albanese E, Launer LJ, Egger M, Prince MJ, Giannakopoulos P, Wolters FJ, et al. Body mass index in midlife and dementia: systematic review and meta-regression analysis of 589,649 men and women followed in longitudinal studies. Alzheimers Dement. 2017;8:165–78. 10.1016/j.dadm.2017.05.007 10.1016/j.dadm.2017.05.007 PMC5520956 28761927

[64] Bastos-Leite AJ, van der Flier WM, van Straaten EC, Staekenborg SS, Scheltens P, Barkhof F. The contribution of medial temporal lobe atrophy and vascular pathology to cognitive impairment in vascular dementia. Stroke. 2007;38:3182–5. 10.1161/strokeaha.107.490102 17962598 10.1161/STROKEAHA.107.490102

[65] Visser PJ, Verhey FR, Hofman PA, Scheltens P, Jolles J. Medial temporal lobe atrophy predicts Alzheimer’s disease in patients with minor cognitive impairment. J. Neurol. Neurosurg. Psychiatry. 2002;72:491–7. 10.1136/jnnp.72.4.491 11909909 10.1136/jnnp.72.4.491 PMC1737837

[66] Chauveau L, Kuhn E, Palix C, Felisatti F, Ourry V, de La Sayette V, et al. Medial temporal lobe subregional atrophy in aging and alzheimer’s disease: a longitudinal study. Front. Aging Neurosci. 2021;13:750154 10.3389/fnagi.2021.750154 34720998 10.3389/fnagi.2021.750154 PMC8554299

